# Lentiviral Vectors Delivered with Biomaterials as Therapeutics for Spinal Cord Injury

**DOI:** 10.3390/cells10082102

**Published:** 2021-08-16

**Authors:** Ciara Shortiss, Linda Howard, Siobhan S. McMahon

**Affiliations:** 1 Anatomy, School of Medicine, College of Medicine Nursing and Health Sciences, National University of Ireland Galway, H91 W5P7 Galway, Ireland; linda.howard@nuigalway.ie; 2 Regenerative Medicine Institute, National University of Ireland Galway, H91 TK33 Galway, Ireland; siobhan.mcmahon@nuigalway.ie

**Keywords:** spinal cord injury, gene therapy, lentiviral vector, bioscaffold, biomaterial

## Abstract

Spinal cord injury (SCI) is a devastating trauma that can cause permanent disability, life-long chronic issues for sufferers and is a big socioeconomic burden. Regenerative medicine aims to overcome injury caused deficits and restore function after SCI through gene therapy and tissue engineering approaches. SCI has a multifaceted pathophysiology. Due to this, producing therapies that target multiple different cellular and molecular mechanisms might prove to be a superior approach in attempts at regeneration. Both biomaterials and nucleic acid delivery via lentiviral vectors (LVs) have proven to promote repair and restoration of function post SCI in animal models. Studies indicate that a combination of biomaterials and LVs is more effective than either approach alone. This review presents studies supporting the use of LVs and LVs delivered with biomaterials in therapies for SCI and summarises methods to combine LVs with biomaterials for SCI treatment. By summarising this knowledge this review aims to demonstrate how LV delivery with biomaterials can augment/compliment both LV and biomaterial therapeutic effects in SCI.

## 1. Introduction

Traumatic spinal cord injury (SCI) is considered one of the most devastating injuries a person can undergo, causing permanent loss of movement and sensation in an instant. It is particularly devastating as it affects young otherwise healthy people with the average age of incidence of 43 years [1]. Due to the loss of motor and sensory functions after SCI, patients also suffer from numerous ‘hidden injuries’. These include neuropathic pain that is experienced by 50–60% of patients and muscle spasticity experienced by 70% of patients [2]. SCI patients also have an increased risk of endocrine, metabolic, nutritional, and nervous system disorders, as well as musculoskeletal and mental health disorders. Other issues include loss of bowel and bladder control, decreased wound healing and pressure sores [3]. SCI significantly reduces the average life expectancy of all sufferers. Life expectancy is reduced by over 10 years on average for paraplegic patients younger than 40 who survive their first year after injury. Life expectancy can be reduced by over 20 years in cervical injury patients who survive a year after injury [1]. SCI also is a big socioeconomic issue. It’s estimated that as few as 35–40% of people with SCI are employed after injury [1,4]. SCI is a life-long condition that is managed conservatively with symptomatic treatment and physical rehabilitation. Due to the complexity of SCI and limited regenerative capacity in the central nervous system, complete recovery of neural function is rare [5]. Research into regenerative therapies for SCI aims to overcome regenerative deficits after injury using gene therapy and tissue engineering to improve neural repair and restore function.

The mechanical forces that cause traumatic SCI are termed the primary injury. These forces can be one or a combination of contusion, compression, distraction or laceration of the spinal cord [6]. These forces cause axon shearing, rupture of blood vessels and immediate cell death. These primary injuries trigger an array of pathological cellular, molecular and biochemical cascades known as the secondary injury that extends the region of damage beyond the impact point. The secondary injury is commonly divided into 3 phases: acute, subacute and chronic. The structure of the lesion changes over these phases, and as the injury progresses the challenges to repair and regeneration accrue (reviewed [7,8,9,10]).

Blood Spinal Cord Barrier (BSCB) breakdown, poor re-vascularisation of the injured area, Wallerian degeneration, demyelination, un-resolving inflammation, scarring and the absence of supporting tissue for axon growth in the injured region are seen as the main obstacles to regeneration after SCI. These processes are prevalent at different time points after the injury, many arising in the early subacute stages, and have a prolonged impact on the course of the injury. [7,8,9,11].

The varying nature and prolonged time span of these obstacles to repair call for strategies that can deliver a sustained impact on multiple obstacles which prevent spinal cord regeneration. Combinatorial regenerative strategies that target multiple aspects of the injury to promote regeneration show the most promise in relieving symptoms and regaining function following SCI [5,12]. Here we highlight how lentiviral vectors (LVs) in combination with biomaterials can provide a long-term impact on the injury with a multidirectional therapeutic approach that targets multiple issues arising after SCI to increase regeneration.

## 2. LVs in SCI Research and Therapies

Gene therapy manipulates the expression of genes to alter a cell’s state, function, or capability for therapeutic benefit via the delivery of exogenous nucleic acids. Altering the expression of different genes allows for greater specificity in molecular targets than with pharmacological interventions. As SCI is chronic in nature, long term sustained delivery of therapeutics to the spinal cord is required. Many therapeutically relevant molecules, particularly proteins, have relatively short half-lives, requiring continuous replenishment over a long period of time to maintain their beneficial effect. This is usually achieved using multiple injections, osmotic pumps or catheters which can greatly increase the risk of infection. Gene therapy can overcome these issues by providing a long term source of therapeutic agent following a single administration [12].

There are viral and non-viral approaches to gene delivery. Non-viral approaches include cationic lipoplexes, dendrimers and naked nucleic acids paired with scaffolds or transfection agents [13]. While non-viral gene delivery strategies may allow larger amounts of nucleic acids to be delivered and have no risk of integration related mutagenesis in comparison to viral vectors, viral vector gene delivery has demonstrated better clinical therapeutic potential due to viral vector efficiency in gene transfer and ability to achieve long term expression *in vivo* [13,14]. Recombinant viral vectors retain the infectious properties of the original virus (enabling them to enter cells) but do not cause diseases and cannot replicate once inside a host cell as their pathogenic genes have been replaced with therapeutic genetic material. Gammaretroviruses, adenoviruses, lentiviruses and adeno-associated viruses have all been studied as therapeutic delivery agents for SCI. This section compares recombinant LVs and adeno-associated viral vectors (AAV) as these are the most commonly used viral vectors in SCI research due to their tropism for neural and glial cells and ability to establish long term transgene expression in non-dividing cells such as neurons [15].

LVs are part of the *Retroviridae* family of viruses. Retroviruses are enveloped viruses with a 9 kb single stranded RNA as their genetic material and have previously been used in clinical trials for indications other than SCI [15,16]. The vesicular stomatitis Indiana virus- G (VSV-G) protein is commonly used to pseudotype LVs, conferring them with a board tropism as it recognises low-density lipoprotein receptor that is expressed on most cells [15]. VSV-G peudotyped LV have been demonstrated to transduce P75+ or S-100+ Schwann cells, Glial Fibrillary Acidic Protein positive (GFAP+) astrocytes, NeuN+ neurons, ED-1+ macrophages and rPH+ fibroblasts in uninjured and injured spinal cords [17,18]. There are claims that VSV-G peudotyped LVs preferentially target glia versus neuronal cells [19]. The ‘third generation’ self-inactivating LVs lose their ability to transcribe viral packaging genes once inserted into the host genome. This reduces the possibility of recombination with another virus that may have infected that cell and is a significant advance in the safety of LV therapies [15,20].

Sustained transgene expression is needed to treat chronic pathological processes, and there is evidence that this improves outcomes in some SCI approaches [21,22]. LV integration into the genome allows for transgene expression potentially for years after a single administration [23]. This is particularly helpful in research settings ensuring sustained efficient expression of the gene of interest compared to transfection and episomal vectors. Insertion of LV cDNA into the host cell genome is necessary for transgene expression. Therefore, insertional mutagenesis is a concern when using LV in a patient population. Insertion is catalysed by the viral integrase enzyme; however integration site choice is attributed to host cellular chromatin readers that are co-opted by the viral integrase. LV cDNA integration site is strongly biased towards actively transcribed genes (with a further preference towards insertion into introns) making the insertion site cell type dependent [24,25]. It is not necessary for AAVs to integrate for gene expression. Transgenes delivered by AAVs predominately exist in the host cell nucleus as non-replicating episomes. However integration into the host genome can occur with AAVs which can facilitate long term gene expression [26,27]. Wild type adeno-associated viruses have a well characterised integration site on chromosome 19 termed the AAVS1 site, which they integrate into during their life cycle’s latent phase. Even though AAVS1 is preferred, integration at other sites in the genome can also occur with wild type adeno-associated viruses. Integration of recombinant AAV genes into AAVS1 and other sites throughout the genome has also been documented [26]. Recombinant AAV integration has been associated with DNA damage, interacting with repair pathways involved in both homologous recombination and non-homologous end-joining. This has led scientists to theorise that recombinant AAV genes integrate ‘‘into the host chromosome as a passive bystander rather than an initiator of recombination’’ [26]. AAV integrations predominantly occur at sites that are highly transcribed thought to be due to the higher occurrence of DNA damage at these sites. Recombinant AAVs integrate at a rate of 0.1–0.5 integrations per infectious unit in human cell culture [26]. For these reasons, although insertional mutagenesis concerns are still present with AVV use, AAVs pose fewer concerns related to insertional mutagenesis than LVs [15,26]. Non-integrating LVs have been developed, in which the vector retains its ability to transduce non-dividing neural cells, yet the genetic material delivered remains as an episome. Similar to episomal transgenes delivered by AAV, this causes transgene expression to be relatively short-lived in dividing cells which may not be desirable in some SCI strategies (e.g., due to cell death after injury and fast proliferation of glial cells upon inflammation) [11,15] but could prove useful for therapies targeting non-dividing spinal cord cells after injury. Strategies are also being developed to allow site specific integration of LVs into the host cell genome, for example co-transduction with Zinc finger nucleases can help to specify the LV integration site [15]. Temporal control over LV transgene expression can be achieved in SCI environment using regulatable gene expression systems such as antibiotic on-off systems [28,29]. These systems could further increase the safety and utility of lentiviral administration to patients. Localisation of a vector to the target area to prevent off-target transductions is a concern for all gene therapy strategies. Even though the transgene may not be expressed in off-target transduced cells (as the promotor is targeted) LV insertion into the genome of off-target cells may cause unwanted effects. Delivery of LV with biomaterials increases LV localisation to delivery site (discussed further below).

LVs have three primary advantages over AAVs. Firstly, pre-existing immunity in the human population to lentiviruses is considerably less than for adeno-associated viruses. Pre-existing immunity causes a strengthened host immune response to viral vector delivery which can pose both safety and efficacy concerns for gene therapy. Adeno-associated viruses are common in human infections. In individuals who have previously been exposed to Adeno-associated viruses, once delivered therapeutic AAVs would not persist long in the body as antibodies against AAVs will inactivate and clear them away. This greatly reduces transduction efficiency and therapeutic effect [30]. The prevalence of neutralising anti-AAV antibodies in the populations differs for different AAV serotypes. Studies suggest that between 4–50% of people have antibodies against AAV5, ~20–100% have antibodies against AAV2 and up to 94% have antibodies against AAV8 [31]. Other AAV serotypes are less prevalent in the human population, however potential pre-existing immunity is an important factor to consider for the feasibility of delivering AAVs as a therapy. Humans are not natural hosts for VSV.G infection, and therefore it is highly unlikely to find specific LV reactive antibodies in the population (although some non-specific cross-reactive antibodies may be present in the population) [32]. Thus, LV gene therapy has a decreased likelihood of an exaggerated inflammatory response to delivery or immune detection and clearance. This would increase LVs transduction efficiency and likelihood of having desired therapeutic effects compared to AAVs in the human population [30]. Secondly LVs have a larger genome than AAVs and therefore capacity to accommodate a larger transgene for insertion (insertion space 7.5–8 kb for in LV versus approximately 5 kb in AAV) allowing a broader range of gene approaches to be used in LVs [30,33,34].

Lastly LVs are reported to have a shorter time frame from delivery to peak transgene expression than AAVs. This is an important consideration in therapeutic use for SCI in which immediate/fast action may be required in the acute injury phase, e.g., to prevent inflammation or take advantage of an early regenerative response [35,36]. Some sources relay the time from delivery to onset of transgene expression from AAVs to be 2 weeks or more [30,37]. This delay is thought to be due to the need for host machinery to synthesise double stranded DNA from AAV single stranded DNA before transcription and expression [30]. However, looking at research using AAV gene therapy in animal models, it may be more accurate to say that peak expression can take over two weeks to occur after AAV delivery. AAV transgene delivery to a mouse spinal cord crush injury saw transgene expression at the earliest time point of 1 week post-delivery with peak expression reached at 4 weeks [38]. Peak transgene expression can occur within approximately 48 h after LV delivery to the central nervous system (with woodchuck regulatory element and human cytomegalovirus promotor) [14,30]. The long time frame from AAV delivery to transgene expression has been called “an obstacle in acute or traumatic conditions, where quick response is needed” [39] making LV delivery a more advantageous choice.

While LV delivery to the spinal cord has benefits over other vectors in SCI treatment, improvements in transgene persistence, localisation, temporal release and decreased immune inactivation can be made by delivering LVs with biomaterials rather than delivery via bolus injection. Below we outline how LVs could be combined with biomaterials to further improve their applicability for SCI therapies.

## 3. Benefits of LV Delivery with Biomaterials

A biomaterial is any structure or substance that is designed to interact with a biological system. Biomaterials can be derived from natural sources or be synthetic. Both natural, synthetic and combined natural-synthetic materials have been used to fabricate biomaterial scaffolds for SCI treatment based on different favourable characteristics that each category has. See Liu et al. [40] for a recent review of how biomaterials have been used on their own or in combination with cell transplants for SCI treatment.

**Figure 1:**
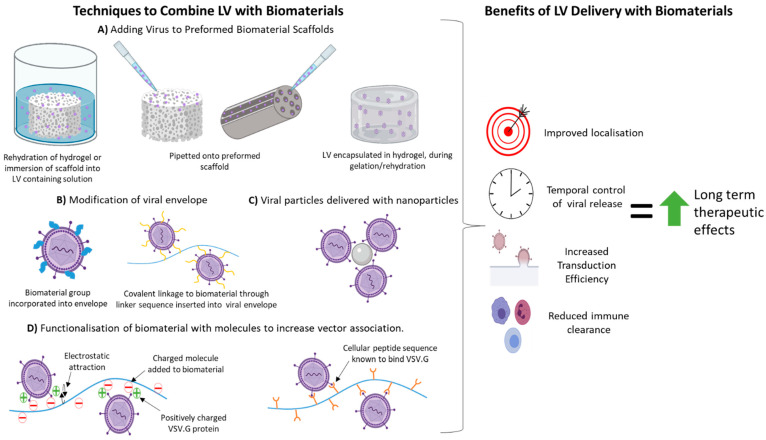
Techniques to Combine Lentiviral Vectors with Biomaterials and the Potential Benefits from such Combinations. (**A**) Lentiviral vector (LVs) particles have been added to biomaterial scaffolds via hydration of the scaffold in LV containing fluid or pipetting LVs directly onto the preformed porous or channel containing scaffolds. LVs can be incorporated into hydrogels prior to gelation. In these cases, LVs associate with the biomaterial in a non-specific manner unless the biomaterial has been modified before scaffold formation. (**B**) The viral envelope can be modified to incorporate a biomaterial molecule, or a molecular linker sequence can be added to allow covalent attachment of viral particle to a biomaterial. (**C**) Nanoparticles can non-covalently associate with LV particles. LV-nanoparticle complexes can be delivered on their own or incorporated into scaffolds pre- or post-formation. (**D**) Functionalisation of a biomaterial either pre- or post-scaffold formation with charged molecules or specific peptide sequences that increase the specific association of viral particles to biomaterials. Combining LVs with biomaterials can improve their localisation to the site of delivery, cause prolonged release of LVs into the environment and decrease immune clearance of LVs. All these effects increase transduction efficiency and transgene expression to enhance the efficacy of the LV therapy. This figure was created with the use of Biorender.com.

Combining LVs with biomaterials has several general advantages for therapeutic transgene delivery all of which increase transduction efficiency to have more potent long term therapeutic effects. Methods used to combine biomaterials with LVs and the benefits associated with LV delivery with a biomaterial are summarised in Figure 1.

LV delivery on a biomaterial provides better spatial control/localisation over LV delivery via bolus injection. When LVs are delivered via bolus injection there is a spatial distance between the vector and its target cell that must be overcome before transduction can occur (mass transport limitations). Attaching LVs to a biomaterial (particularly one that is favourable to cell adhesion) puts the vector directly into the cell microenvironment, thus greatly improving their ability to transduce cells. The extent of cell transduction following LV delivery on fibrin scaffolds has been found to be directly dependent on the extent of cell- fibrin interactions [41]. Delivery of LV on a biomaterial also decreases viral diffusion away from the site of delivery. This decreases the potential for off-target effects while increasing the local concentration of virus, probability of cell-LV interaction and thus transduction efficiency. Wu et al. found that greater transgene expression could be achieved with half as many LV particles when LVs were delivered with a Pluronic F127 hydrogel than when LVs were delivered via bolus injection to a rat transection SCI [42]. LVs can be attached to biomaterials so that they will remain immobile on the scaffold surface and are not released into the surrounding environment. This spatially restricts transduction to only cells that infiltrate the scaffold [17,43]. Vectors can also be combined with biomaterials so that they are gradually released into the adjacent tissue as the biomaterial/LV-tethering component degrades [17,44,45,46,47]. This increases the concentration locally so an increase in transduction efficiency is found but does not limit transduction to only cells that infiltrate the scaffold. When LVs were attached to poly (lactide-co-glycolide) (PLG) via a phosphatidylserine (PS) molecular tether added during scaffold formation and these LV-loaded scaffolds were implanted into a rat spinal cord hemisection, maximal transgene expression was found at the site of implant/injury and decreased with distance from implant site. Spinal segments directly adjacent to the implantation site had 4–6 fold less transgene expression, and in segments further away than adjacent segments the level was only 6% of the maximal expression [48]. Tuinstra et al. 2012 [18] found creating LV hydroxyapatite (HA) nanoparticle (NP) complexes and then loading them into the preformed channels of a PLG scaffold provided localised transgene expression after implantation into a rat spinal cord hemi-section model. Transgene expression was highest at the center of the implant with good cell ingrowth. 90% of total transgene expression was in the implant and directly adjacent segments (1 cm rostrally and caudally) at 1 week post implant. This region contained 80% of the total transgene expression at 4 weeks. The most distant transgene expression noted was 2.5 cm rostrally and caudally from the implantation at both 1 and 4 weeks post implant. No comparison was made with bolus injection or with LV loaded PLG scaffold alone [18].

The temporal release of LVs into surrounding tissue can also be adjusted depending on the biomaterial and on the method used to attach the LVs. Collagen and chitosan/b-glycerol phosphate (chitosan BGP) hydrogels were found to have different LV release profiles in an *in vitro* setting over a three day time period [49]. 0.1% weight per volume (*w*/*v*) collagen scaffolds released the highest number of LVs on day 1 of the study with negligible active LVs release on day 2 and 3. In total over three days collagen scaffolds released less active LVs than scaffolds made from any concentration of chitosan BGP (3.12%, 2.38% and 2.17% *w*/*v*). Chitosan BGP hydrogels prolonged the release of LVs, with 2.17% chitosan scaffolds releasing a similar level of active LVs on days 1 and 2 and then an increased amount on day 3 of the *in vitro* study. With increasing concentration of chitosan BGP the total amount of virus released over time decreased. This effect was found to be independent of scaffold pore size [49].

LVs can be combined with biomaterials using several different methods which also influence their retention and release at the site of implantation. LVs pipetted onto unmodified PLG were all released after 3 days incubation at 20 °C in PBS. Coating the PLG with chitosan or heparin molecules retained >40% of LVs and ~100% of LVs respectively under the same conditions [46]. Stilhano et al. [50] found that with higher molecular weight alginate (250 kDA) gels degraded more slowly over time *in vitro* (in cell culture media at 37 °C and 5% CO_2_) in comparison to lower molecular weight alginate (50 kDa) gels resulting in more prolonged LV release. 75%/25% low/high molecular weight gels released ~65% of LVs over the 6 day elution study, whereas 25%/75% low/high molecular weight gels released ~45% of LVs and 100% high molecular weight gels released ~15% LVs [50]. For more information on LV retention and release see Section 4 below on techniques used to combine LVs with biomaterials.

Combining LVs with biomaterials also improves viral stability and half-life which further increases transduction efficiency. LV half-life and stability are also affected by the LV-biomaterial attachment method. The half-life of LV combined with unmodified polyethylene (glycol) (PEG) scaffolds in cell culture media at 37 °C and 5% CO_2_ was 8.3 h compared to 10 h on high molecular weight poly-L-lysine (PLL) modified PEG scaffolds [51]. Chitosan and heparin coated PLG extended LV half-life to 41.5 and 40.4 h, respectively, vs 20.8 h on unmodified PLG scaffolds in PBS at room temperature [46]. Shin et al. [44] mixed LV with HA NPs to create LV-NP complexes. After incubating LV-NPs and LVs alone in PBS at room temperature for different lengths of time LV-NP complexes resulted in a 2.9-fold increase in transgene expression in target cells than LVs incubated in PBS solution alone [44].

The increased transduction efficiency of LVs from delivery on a biomaterial due to the above advantages causes transgene expression to be more potent and to be sustained for longer periods of time. LV transduction alone provides long term transgene expression once cells have been transduced. Prolonged or sustained transgene expression in a SCI environment with substantial cell turnover could be caused by either (i) an increased number of cells initially transduced (transduction efficiency being improved by localisation) or (ii) by new cells continuously being transduced over a longer period of time (transduction efficiency being improved by LV half-life, persistence or gradual release). As some pathological processes span months to years in SCI, prolonged release and the increased persistence of therapeutic LV/transgene expression that LV delivery with biomaterial allows may be desirable over LV delivery alone [5]. When injected into mouse skeletal muscle LV-alginate injections maintained transgene expression for 2 weeks longer than bolus LV injections (49 days prolonged to 77 days until the end of study) [50]. Loading LVs onto a PLG scaffold with HA nanoparticles incorporated into the scaffold produced a higher initial transgene expression after epididymal fat pad implantation when compared to LVs loaded onto PLG scaffolds alone or LV-HA NP complexes delivered on their own [52]. This initial increased expression decreased and after the first week and all scaffolds had similar levels of transgene expression. However transgene expression from the latter control groups fell below background at 50 days while animals with LV loaded PLG scaffolds with HA-NPs maintained a steady state, significantly higher level of transgene expression until 100 days (end of study) time point [52]. LVs delivered on heparin or chitosan coated PLG scaffolds implantated into a mouse SCI hemi section model caused significantly greater transgene expression from 17 days and 38 days onwards respectively, in comparison to LV loaded unmodified scaffolds. Peak transgene expression from modified scaffolds was 3.9-fold and 2.7-fold greater than unmodified scaffolds for heparin and chitosan coated bridges, respectively [52]. Similarly, LVs loaded on to a PEG hydrogel with heparin-chitosan nanoparticles within it (at a 3:1 ratio) maintained transgene expression for longer time periods after subcutaneous implantation in mice. For the initial two weeks post implant LV loaded unmodified hydrogels had a lower level of transgene expression than LV loaded PEG hydrogels without NPs. From 28 days onwards LV loaded scaffold containing the heparin-chitosan NPs caused a higher level of expression and maintained this for the 56 day duration of the study [45].

Due to disruption of the BSCB after SCI the LVs may be exposed to all circulatory innate (complement system) and adaptive (antibody driven) anti-viral responses which can decrease their persistence and reduce transduction efficiency [53]. Pre-existing immune responses to LVs are unlikely in humans, however non-specific cross-reacting neutralising antibodies to the VSV.G pseudo-envelope protein, LV matrix protein P17 and LV capsid protein P24 have been found to hamper LV efficacy after systemic injection [54]. Membrane proteins from the host cell used for LV generation can also be incorporated in the vector envelope and also trigger immune reaction, and therefore must also be considered and accounted for when producing LV therapies for patients, e.g., MLA1 class negative producing cell lines [15]. Biomaterials can ‘shield’ LVs from the immune system and therefore immune mediated inactivation and clearance [53]. Croyle et al. [53] conjugated PEG polymer molecules to the LV envelope with the aim of decreasing viral recognition and clearance by the complement immune system. PEGs were added by mixing 10 g of PEG polymer per µg of LV at 25 °C with gentle stirring. After 1 h there were 3000 PEG molecules coupled to each virus particle (determined by ELISA). PEGylated and unPEGylated LVs were added to 293 T cells in the presence of serum containing neutralising antibodies against unmodified LV-VSV.G (VSV.G-AB) and human serum with normal complement levels. Transduction efficiency of unPEGylated LVs decreased with VSV.G-AB serum conc. (94% decrease in transduction efficiency compared to normal culture conditions). PEGylated LVs were not affected by VSV.G-AB serum concentration. After exposure to human serum with complement proteins unPEGylated and PEGylated LVs retained an average of 29%, and 79% of their original activity, respectively. After exposure to mouse serum PEGylated LVs were unaffected whereas unPEGylated reduced to 20% of original activity. LVs were then injected into the tail vein of mice to evaluate immune clearance and transduction *in vivo*. Two weeks after tail injection a three- and six-fold higher amount of transgene expression was found in the bone marrow and spleen, respectively, of mice injected with PEGylated vector in comparison to unPEGylated vector. Animals treated with the PEGylated LVs also had a significantly lower level of aspartate transaminase and alanine transaminase (indicators of liver damage) than animals given unmodified virus [55].

Biomaterials can also be leveraged to alter cell infiltration (as different cells have different affinities for different biomaterials) and thereby influence the cells transduced by LVs. Boehler et al. [52] found that after implantation of LV containing PLG scaffolds into the epididymal fat pad of male mice 60% of transduced cells were identified as CD45+ immune cells at day 3, and this proportion remained the same on day 21. For PLG scaffolds containing HA NPs (PLG/HA-NP) approximately 60% of transduced cells were also identified as CD45+ at day 3 however at day 21 only 25% of cells expressing the transgene were CD45+, 75% were non-immune CD45- cells. The total number of immune cells in PLG/HA-NP scaffolds was also reduced compared to the unmodified PLG scaffold on day 21 [52]. The direct tropism of viral vectors has been modified by the addition of biomaterials to their capsid/envelope (AAV modified vectors reviewed in [56]). At the time of writing this review to my knowledge altering LV tropism by directly attaching biomaterials to the virion has not been studied, yet the success of this method when applied to other viral vectors could be adapted to LVs in the future.

Biomaterials have inherent properties valuable for SCI treatment. Many studies have used biomaterials alone as a treatment for SCI (reviewed [40]). Biomaterials can provide granulation tissue with a surface matrix to encourage ingrowth of cells and axons after injury. In chronic SCI in particular the cystic cavity provides no structural support for cellular repopulation and potential regeneration. Therefore removal of ‘cavity’ tissue and replacement with a biomaterial scaffold consisting of fibres or channels is a strategy of interest, as it provides structural stability to the injury as well as an inductive substrate to encourage axon and glial ingrowth (reviewed [57]). In acute SCI hydrogels have been suggested to help seal the dura if compromised by injury [5] while hydroxyapatite nanoparticles have been found to decrease haemorrhage and oedema after spinal cord stretch injury [58]. Targeting these aspects in acute SCI could help limit the secondary injury and the obstacles to regeneration it presents. Aside from their beneficial structural support, biomaterials can also evoke biological responses. Biomaterials with inherent immunomodulatory (reviewed [59]) angiogenic (reviewed [36]) electro-physio-chemical (to help with external stimulation rehabilitation in SCI [57]), piezoelectric (e.g., Poly-β-hydroxybutyrate [60]) and membrane sealing (e.g., PEG [61], chitosan [62]) properties have been studied in the SCI context.

Delivering LVs on a biomaterial combines the biomaterial’s beneficial properties with LV therapy while delivering and enhancing the efficacy of the LV therapy. This combination could be synergistic, targeting one or more obstacles to regeneration after SCI. Due to these advantages treatment with LVs delivered with biomaterials has resulted in improved functional outcomes in animals’ models of SCI than those treated with either therapy on their own (see Section 5 below on ‘Studies combining LVs with Biomaterials as Regenerative Therapies for SCI’).

## 4. Techniques Used to Combine LVs with Biomaterials Previously Used in SCI Regeneration Therapies

As outlined above LV delivery with biomaterials can be beneficial for many reasons. The affinity of interaction between the biomaterial and LV is essential for vector retention, stability and in turn transduction efficiency. These properties are influenced by the mechanism of interaction or attachment of vector to biomaterial. The specific technique used to attach/combine a LV with a biomaterial can also have advantages and disadvantages associated with it in relation to LV transduction efficacy and applicability to SCI therapies. Discussed below are techniques which have been used to combine LVs with biomaterials that have previously been used in SCI therapeutic applications. Table 1, Table 2, Table 3, Table 4 and Table 5 summarise studies on how these attachment methods affect LV retention, half-life, transduction efficiency and other factors in a range of different *in vivo* and *in vitro* models. Even though some of these studies are *in vitro*, or do not use SCI as the *in vivo* model and therefore may not be directly relatable to a SCI environment, all biomaterials mentioned have previously been studied as a therapy in SCI (with or without LVs) and information on the LV-biomaterial pairing and attachment method’s influence over LV dynamics may prove beneficial for application to SCI therapeutics.

### 4.1. Non-Specific Binding/Assocaition with Biomaterial

This method of LV immobilisation relies on electrostatic interaction, van der Waals forces and hydrophobic interactions between biomaterial and vector. These are usually achieved by incubating the LVs in contact with the material, mixing or lyophilisation of LV with biomaterial to cause direct adsorption [63]. This method is simple and does not require specialised equipment to modify the biomaterial. One key advantage of this approach is that it usually doesn’t expose the LV to extreme temperatures/pressures used in the biomaterial production process that could damage or destroy it. Non-specific association is dependent on the natural affinity of LVs to bind the biomaterial through non-covalent bonding and thus may not always provide optimal virus retention and release rates. Studies using non-specific binding to biomaterials show, long term transgene expression, some transgene expression localisation and improvement in viral transduction [44]. However non-specific binding is not enough to completely localise transgene expression to the site of implantation. This suggests that the biomaterial is preventing some vector clearance and increasing transduction after delivery even if not all vector is retained at the implant site. Studies delivering LVs on a biomaterial using non-specific binding are summarised in Table 1.

**Table 1:** Studies using non-specific binding/association for LV delivery on biomaterials. PLG; poly (lactide-co-glycolide), CL; Poly (ε-caprolactone), PEG; Polyethylene (glycol).

Non-Specific Binding/Association with Biomaterial				
Biomaterial	Attachment Details	Model	Vector Retention and Transduction Efficiency	Ref
PLG	Lyophilisation with sucrose.	HEK cells. Subcutaneous implantation in mice.	Total immobilised LV on scaffold was low. >80% of virus was released within 24 h *in vitro*. Transduction efficiency of LV 1.8 times greater than bolus delivery *in vitro*. Transgene expression confined to disc area after subcutaneous transplantation (no comparison to bolus).	[63]
PEG or Gelatin	Pipetted into channels.	Intrathecal delivery to uninjured and thoracic spinal cord hemi-section injury in mice.	60% and 25% of transgene expression was localised to uninjured spinal cord at 2 weeks, from PEG and gelatin scaffold, respectively. Transgene expression persisted for 12 weeks, declining after 9 weeks on both scaffolds, with 14% greater expression in PEG over gelatin scaffold across time (no comparison to bolus delivery). Transgene expression when LV-PEG was delivered immediately after injury was almost 3-fold greater than when delivered 4 weeks after injury.	[17]
PCL	Pipetted onto scaffold and incubated 2 min before implantation.	Implantation into mouse periovarian fat pad.	Peak transgene expression 7 days post implantation; expression maintained for 56 days. Low level of transgene expression observed throughout mouse trunk. Highest expression around implant site.	[64]
PEG	PEG-mal hydrogel tubes with LV injected directly into tubes before implantation.	Mouse C5 1.15 mm lateral hemisection.	Transgene expression peaked at 4 weeks post implant with decline but significantly higher level of expression over background until 12 weeks end of study.	[65]

### 4.2. Surface Modification/Functionaliseation of Biomaterial

Functionalisation/coating of the biomaterial aims to increase non-specific binding (as outlined above) or direct (covalent) binding of LVs to scaffolds. Addition of common polysaccharides [46] and peptides [47,51,64,66] as well as the incorporation of LV-targeted peptide sequences that capitalise on innate LV binding to cells [48,51] into biomaterials have been studied. Studies that have added moieties/functionalised a biomaterial to aid with LV delivery are summarised in Table 2.

Adding moieties that associate with LVs provides higher, more controllable levels of LV retention and release in comparison to direct adsorption onto biomaterial via non-specific binding. LVs are generally added after modification and therefore are not exposed to production process. Addition of moieties to scaffolds can alter their physical and mechanical properties. Surfaces modifications aim to minimise changes to scaffold architecture, mechanics and bulk properties of the material while still providing a more suitable substrate for LV attachment [46].

The VSV.G envelope protein has a cluster of positively charged amino acids in its structure that are thought to interact with the plasma membrane receptor of phosphatidylserine and mediate internalisation of the LV [67]. Similar ionic interactions could conjugate LVs to negatively charged molecules such as heparin. Addition of ionic groups may also facilitate the adhesion of cells to biomaterials further leading to greater association between cells and LVs resulting in increased transduction [46]. Positively charged molecules, e.g., chitosan [46] and poly-L-lysine [51] have been found to increase viral half-life and efficiency. This is thought to be through shielding of repulsion between the negatively charged viral coat and the negatively charged cell membrane enhancing their association and therefore transduction [46]. Not all negatively charged molecules (e.g., hyaluronan) improve vector retention, indicating that charge is not the only factor influencing transduction [68].

A common way to attach molecules to biomaterials is through the formation of an amide bond. Many natural and synthetic biomaterials have carboxyl groups and many molecules used to functionalise scaffolds have amine groups. Carboxyl-reactive chemicals such as N-ethyl-N′-(3-(dimethylaminopropyl) carbodiimide (EDC) are used to activate carboxyl groups. Other chemicals such as N-hydroxysuccinimide (NHS) can be added to stabilise intermediary amine-reactive ester groups. These chemicals catalyse biomaterial carboxyl group reaction with polysaccharide amine groups creating an amide bond that covalently links a molecule to the a biomaterial [69].

The biomaterial-moiety pairing and the way in which moieties are incorporated into/attached to the biomaterial also affects LV release and transduction efficiency. Thomas et al. [46] found increased LV transgene expression in chitosan and/or heparin coated PLG linked through EDC/NHS chemistry when delivered to a SCI model. Further studies by Thomas et al. [45] found LV loaded chitosan/heparin modified gelatin hydrogels created using cysteine cross-linkers and EDC/NHS chemistry, implanted into mouse intrathecal space showed significantly reduced transgene expression in comparison to LV-loaded unmodified gelatin hydrogels. However when heparin was simply mixed with PEG polymer before hydrogel formation, or adsorbed onto PEG by adding heparin after hydrogel formation, these combinations produced significantly higher transgene expression in culture [45]. This result was likely due to heparin increasing LV half-life. The difference in transduction efficiency between these studies is likely due to the release of LVs from biomatierals and the ability of cells to infiltrate the scaffolds in different models which should be considered when choosing a LV attachment strategy in line with the LV therapy.

These studies show that the moiety added, method of its addition and the moiety-biomaterial pairing all impact LV retention/release and transduction efficacy. Surface modifications/the addition of moieties to biomaterials do not have a ‘one size fits all’ effect and the method used to add molecules to the surface of a biomaterial should be investigated to see which yields the desired effect within a particular moiety-biomaterial pairing and LV therapy. These studies also highlight that retention of LVs within a scaffold will only cause the transduction of cells that invade that scaffold and some vector release over time may prove more useful for some therapies.

**Table 2:** Studies using surface modification/ functionalisation for LV delivery on biomaterials. PLG; poly(lactide-co-glycolide), PEG; Polyethylene (glycol), PEGDA; Polyethylene (glycol) diacrylate, PLL; poly-l-lysine, PS; phosphatidylserine, MW; Molecular Weight, SC; spinal cord.

Surface Modification/Functionalisation of Biomaterials				
Biomaterial	Attachment Details	Model	Vector Retention and Transduction Efficiency	Ref
PLG	PS coated PLG microspheres formed into scaffold. LV pipetted onto scaffold before implantation.	Rat SC hemi-section model (spinal segment not given).	PS coating of PLG caused localised transgene expression.	[48]
	Chitosan or heparin immobilised onto PLG post fabrication using EDC/NHS chemistry. Virus pipetted onto modified/unmodified scaffold.	Implanted into mouse T9–T10 SC hemi-section lesion.	Heparin and chitosan coating increased LV incorporation and doubled LV half-life. Transgene expression significantly greater in heparin and chitosan coated bridges. Heparin coated scaffolds maintained the highest transgene expression across the 59 day study.	[46]
Gelatin	Cysteine added as a cross linker to chitosan and heparin in EDC/NHS solution. For hydrogel incorporation, filtered solutions were flash frozen in nitrogen and lyophilised. Virus pipetted onto modified/unmodified hydrogel.	Implantation into mouse intrathecal space above thoracic spinal cord.	Significantly reduced transgene expression from heparin/ chitosan modified -vs-unmodified scaffolds, due to viral retention in scaffold.	[17]
PEG	Low (1–10 kDa) and high (30–70 kDa) MW PLL were added to PEG acrylate hydrogels. Subsequently hydrogel incubated with virus solution.	HT1080 cells cultured on LV containing scaffolds functionalised with PLL of different MW.	Increasing MW of PLL increases virus adsorption to PEG-PLL scaffold. Incubation time of LV with PLL functionalised PEG scaffold effects the extent of virus adsorption. PLL-functionalisation of PEG increases viral half-life.	[51]
	Peptides sequences that bind VSV-G protein. Peptides incubated with virus first then this mixture attached to PEG hydrogel via acrylate—PEG—maleimide linker.	HT1080 cells added to LV containing peptide functionalised scaffolds.	PEG with VSV.G binding peptides attached increases LV binding to levels similar to PEG functionalisation with high MW PLL.	[51]
PEGDA	Non-covalent attachment of PLL to premade PEGDA cyrogel through emulsion in PLL solution. Covalent attachment of PLL to PEGDA through poly-acrylic linker and EDC-NHS chemistry. LV pipetted onto pre-made scaffolds.	Non-invasive NIHT3T3 cells seeded on scaffold and stained to test cell adhesion. Subcutaneous implantation of scaffolds in mice.	PEGDA with covalently linked PLL retained LVs better than scaffolds with adsorbed PLL *in vitro*. Significantly higher *in vivo* cell transduction with covalent PEGDA—PLL scaffold than bolus injection of virus near same scaffold.	[47]

### 4.3. Encapsulation of LVs within Hydrogels

Encapsulation within a hydrogel traps LVs localising their delivery and shielding them from immune clearance. Studies utilising LV delivery with a hydrogel biomaterial are summarised in Table 3. Encapsulation of LVs in hydrogels can expose virus to processing conditions that can impact its activity (e.g., collagen hydrogel gelation at 37 °C for 30 min [44,70]) although hydrogel processing is usually milder than other scaffold formation techniques (e.g., electrospinning, high temperatures and exposure to high-frequency light). Without hydrogel modification the release of viral vectors from hydrogels is determined by the percentage solid in the hydrogel, hydrophilicity, crosslinking and degradation [41,44,49,71]. The microstructure of the hydrogel also impacts LV transduction efficiency by permitting/hindering cell invasion. Strong early cell invasion is preferable to allow cell transduction to occur before LVs are degraded or become inactive [72].

A disadvantage of hydrogel delivery of LVs is their fast degradation rate due to high water content therefore the scaffold that holds cells and vector in close proximity is lost decreasing transduction efficiency. Direct interaction between viral capsid/envelope and hydrogel can decrease the diffusion rate of LVs as the matrix degrades thus increasing local vector concentration and transduction efficiency. The vector and the biomaterial can both be modified prior to combination/encapsulation to increase specific or non-specific interactions. Some hydrogels have a chemical composition and hierarchal structure upon gelation that naturally aids viral retention and prolongs vector release, e.g., Pluronic acid gels have a polyoxypropylene core and hydrated polyoxyethylene chain shell micelle structure [42].

**Table 3:** Studies using encapsulation of LV within a hydrogel for delivery. Chitosan BGP; chitosan/b-glycerol phosphate, EG; Polyethylene (glycol), HyA; Hyaluronic Acid, PLL; poly-l-lysine, MW; Molecular Weight.

Encapsulation of LVs within Hydrogels				
Biomaterial	Attachment Details	Model	Vector Retention and Transduction Efficiency	Ref
PEG	Macroporous PEG hydrogel encapsulating gelatin microspheres. Microspheres were hydrated with LV containing solution before addition to PEG and gelation.	Subcutaneous implant in CD1 mice.	Inclusion of LV loaded gelatin microspheres within a macroporous PEG hydrogel enhanced cell infiltration and sustained transduction at higher levels for longer than macroporous gels without microspheres, and macroporous PEG gels with gelatin microsphere that were loaded with LVs post gelation.	[71]
Collagen	LVs mixed with collagen during gelation.	C6 cells were seeded on gels containing LVs.	Increasing % collagen in hydrogel increases viral stability but can limit cell infiltration and thus transduction. Transduction on all collagen gels regardless of % collagen was ~80% of control transduction efficacy (bolus addition to culture dish).	[44]
Collagen –vs- Chitosan/b-glycerol phosphate	LVs mixed with chitosan BGP or collagen before gelation.	LV elution measured following incubation of scaffolds in cell culture medium.	Different elution profiles for chitosan vs. collagen scaffolds. 2.17% chitosan scaffold provided a more prolonged release of LVs than collagen. Increased concentration of chitosan decreased the amount of LVs released over time.	[49]
Fibrin with/without Polybrene	LVs mixed with thrombin before mixing with fibrinogen (between 3.75–7.5 mg/mL). Polybrene added to some gels before gelation.	LV loaded fibrin gels spotted in a pattern and cells grown on top. Or NIH-3T3 cells or 293T cells seeded on top gels.	Higher transduction efficiency and transgene expression from LV loaded fibrin gels over LV delivered to cells by bolus. High percentage fibrin gels (up to 30 mg/mL) prevented LV elution, with lower percentages (1.5–7.5 mg/mL) yielding the best transduction. Fibrin degradation by target cells may be necessary for successful gene delivery. Polybrene enhances transduction efficiency of LV loaded fibrin gels.	[41]
Alginate	Different ratios of low and high MW (LMW and HMW) alginate polymers (75/25 and 25/75 low/high MW), as well as high MW alginate alone were used to create gels.	LV loaded gels injected into left hind limb muscle of mice.	Concentration of alginate effects virus elution over time. Gels with a higher concentration of LMW alginate have faster elution rates. LV delivery with 75% HMW alginate led to a sustained level of transgene expression for more than two months *in vivo*.	[50]
HyA	3 scaffolds compared: (i) NP-HyA; HyA microspheres (with a thiol group) mixed with RGD-conjugated PEG to form nanoporous HyA hydrogel once in situ. (ii) Mac-HyA; NP-HyA hydrogels crosslinked around PEG microparticles that proteolytically degrade in situ to leave behind a macroporous architecture. (iii) HyA-MP; polydisperse HyA-PEG microparticles (from NP-HyA scaffold) assembled in situ. Precursor molecules were mixed with PLL and LVs before injection and gelation *in vivo*.	Injected into mouse left/right mammary fat pad with opposite pad acting as internal LV loaded NP-HyA control.	HyA-MP hydrogel had ~8-fold greater cell density and 16-fold greater transgene expression in comparison to NP-HyA and Mac-HyA scaffolds.	[73]
	3 scaffolds compared: (i) NP-HyA; HyA microspheres (with a thiol group) mixed with RGD-conjugated PEG to form nanoporous HyA hydrogels once in situ. (ii) Monodisperse HyA-PEG (mHyA-MP) or (iii) polydisperse (pHyA-MP) microparticles assembled in situ. Precursor molecules mixed with PLL and LVs before injection and gelation *in vivo*.	Injected after spinal cord T8-T10 clip compression injury in mice.	Transgene expression as far as 1.5 mm from site of injection. mHyA-MP had significantly increased expression at centre of scaffold in comparison to pHyA-MP and NP-HyA scaffolds.	[72]

### 4.4. Modification of LV Envelope

LV can be covalently tethered to the scaffold through the addition of moieties to the envelope [43].This allows highly localised and spatially controlled retention of the virus. It also can allow for specific release of the virus upon addition of an enzyme or factor to break the covalent link/tether. However, modification of the LV envelope can reduce its transduction ability. Direct linking of the VSV.G protein to biomaterial was found to also cause steric hindrance reducing transduction. This steric hindrance was relieved with addition of a linker peptide [51]. Modification of the viral envelope with biomaterials can also decrease immune clearance of LVs. Studies altering the LV envelope with biomaterials or envelope alteration to improve LV attachment to a biomaterial are summarised in Table 4.

**Table 4:** Studies that have modified the LV envelope with a biomaterial, or to improve attachment of LV to a biomaterial. aa; amino acid, VSV.G; Vesicular stomatitis virus G PEG; Polyethylene (glycol), CCPEG; cyanuric chloride monomethoxy polyethylene glycol, SSPEG; suc- cinimidyl succinate monomethoxy polyethylene glycol, FXIII; Transglutaminase Factor XIII.

Modification of LV Envelope				
Strategy	Attachment Details	Model	Viral Transduction Efficacy and Retention	Ref
PEG conjugated to VSV.G envelope	LV was conjugated with SSPEG and CCPEG, respectively. 10 g of SSPEG or CCPEG polymer (activated by succinimidyl succinate) was added per µg of protein content in LV preparation. Conjugation reactions were performed at 25 °C with gentle stirring. Reactions were stopped by addition of 103 L-lysine.	PEGylated and unPEGylated LV were added to 293T cells in the presence of serum containing neutralising antibodies against unmodified LV-VSV.G and human serum with normal complement levels. Injection into tail vein of mice for bio-distribution.	Addition of PEG to LVs did not affect transduction efficiency *in vitro* and *in vivo*, and protected the vector from inactivation in complement-active human Serum. Addition of PEG to LVs affected bio-distribution in circulatory system and was less damaging to liver.	[53,55]
Fibrinogen binding site inserted into VSV.G envelope	Introduction of FXIII recognition sequence and protease recognition sites into LV envelope protein sequence. This was achieved by inserting a 17 aa peptide sequence into pMD2.g plasmid (FXIII-LVs). Subsequent incubation of FXIII-LV with thrombin, Ca^2+^ and fibrinogen created a bridge between, FXIII and fibrin, covalently attaching LV to fibrin hydrogels before gelation.	Fibrin gel spots with wild type LVs or FXIII-LVs were printed onto tissue culture slides and a confluent layer of 293 T cells grown on top.	Protease release site in FXIII envelope linker is necessary to maintain infectivity. Enzymatic conjugation of FXIII-LV enables highly spatially controlled gene delivery. FXIII-LV (with protease site) did not significantly alter LV infectivity and significantly reduced release of virus from fibrin gels.	[43]

### 4.5. Use of Nanoparticels in LV Delivery

Modification of biomaterial scaffolds with large molecules can be challenging due to their size and subsequent impact on scaffold physical and mechanical properties (e.g., stiffness, swelling, porosity, cellular infiltration). Due to their size, nanoparticles (NPs) do not impact overall mechanical properties of the biomaterial at the correct concentrations while retaining any innate beneficial LV binding [44]. Most studies involving NPs rely on innate non-specific attraction or adsorption to LVs, yet NP incorporation into biomaterial scaffolds proves to provide better retention than LV adsorption onto a scaffold alone or encapsulation in hydrogel alone, potentially due to the increase in surface area for LV attachment [44,52]. NPs used for LV immobilisation are usually negatively charged, capitalising on the LV’s attraction to negatively charged particles for retention and prolonged release. [18,44,45,52].

NPs have been used as delivery vehicles for LVs in a few ways. One strategy is to mix LVs with NPs creating LV-NP complexes. These complexes have then been delivered on their own [45], loaded onto pre-made scaffolds [18] or encapsulated in hydrogels [44]. Hydroxyapatite NPs (HA NPs) have been incorporated into scaffolds during scaffold formation, and subsequently LV is loaded onto the scaffold [52]. The latter approach does not expose LV or LV-NP complexes to any scaffold formation and thus ensures maximal viral activity is retained. Incorporation of HA-NPs has also been found to influence the speed and type of infiltrating cells [52]. Studies utilising nanoparticles in LV biomaterial delivery are summarised in Table 5. 

**Table 5:** Studies delivering LV in combination with Nanoparticles. NPs; Nanoparticles, HA; Hydroxyapatite, PLG; poly (lactide-co-glycolide) HCNPs; heparin-chitosan nanoparticles.

Use of Nanoparticles in LV Delivery				
Biomaterial	Attachment Method	Model	Vector Retention and Transduction Efficiency	Ref
HA NP + collagen hydrogel.	LVs mixed with HA NPs in PBS then added to collagen before gelation.	Subcutaneous implantation into mouse.	LV combination with HA NPs sustains viral activity for longer periods of time than LV alone when incubated in PBS. LV immobilisation onto HA NPs in a collagen hydrogel provided significantly higher levels of local transgene expression after subcutaneous implantation.	[44]
HA NP + PLG scaffolds	LVs mixed with HA NPs in PBS then loaded into channels of preformed PLG channel-bridge scaffold.	Implanted into Rat SC hemi-section T9-T10.	LV transgene activity was maintained for 4 weeks post SCI implantation. All transgene expression observed within 2.5 cm rostrally and caudally from scaffold. No bolus LV injection control, no LV on PLG only control.	[18]
	LVs pipetted on top of pre-made HA-NP/PLG scaffold compared to LVs mixed with HA NPs and LVs pipetted on top of PLG only scaffolds.	Implantation into mouse epididymal fat pad.	Increased transgene expression, and transgene expression for longer times in comparison to LV loaded PLG scaffold without NPs and LV HA NP complexes implanted without a scaffold.	[52]
Fibrin alone Or fibrin + HA-NP	LVs mixed HA NP. LV-NP complexes mixed with fibrinogen and thrombin solution prior to gelation.	LV loaded gels implanted subcutaneously into mice.	HA slowed fibrin gel enzymatic degradation. Expression levels from fibrin/HA-NP gels were significantly higher than fibrin alone gels from 2 weeks onwards.	[74]
PEG hydrogel functionalised with HCNPs	PEG gels with HCNPs (3:1 heparin: chitosan) incorporated into PEG gels during formation. LVs pipetted on top premade hydrogels.	Subcutaneous implantation in mice.	LV incorporation into hydrogels was significantly improved by incorporating HCNPs. Reduced amount of LVs released from hydrogels with NPs. NP functionalised PEG hydrogels resulted in higher transgene expression declining at a slower rate than control hydrogels *in vivo*.	[45]

## 5. Studies Combining LVs with Biomaterials as Regenerative Therapies for SCI

Therapeutically, there are only a handful of studies that have combined LVs with biomaterials in SCI. The main findings of these studies on both natural and synthetic biomaterials are summarised in Table 6 and Table 7, respectively. Most studies found that the combination of LVs with biomaterials had indications of superior regeneration than biomaterial scaffolds or LVs delivered alone. This is likely due to the increased transduction [42] provided by scaffold localisation, augmentation of LV therapeutic effects [18,75,76] or the addition of another therapeutic avenue ([76,77]).

The speed of host cell infiltration is key in LV delivery on biomaterials. LVs have a relatively short half-life at body temperature (although this may be prolonged with biomaterial addition [44,46,51]) therefore rapid cell infiltration is needed for most efficient biomaterial-mediated delivery of LV transgenes. The pore size and overall porosity of the scaffold has been found to be key in this respect [72]. Other important considerations when choosing a scaffold to combine with LVs include the scarring response, natural versus synthetic biomaterial, preference towards type of infiltrating cell and scaffold implantation versus injection. For a recent review of biomaterials applied therapeutically to SCI see [40].

## 6. Conclusions

There is a growing body of work on the beneficial properties of viral vector delivery with biomaterials for SCI treatment [12,40]. LV delivery using biomaterials and/or LV-biomaterial combinational therapies can work synergistically leading to superior improvement in regeneration markers after SCI. In this review we summarise the strategies used to combine LVs with biomaterials that have previously been trialled in SCI in the hopes of encouraging more studies on the same in search of more effective restoration of function after SCI.

## Figures and Tables

**Table 6:** Studies combining LVs with natural biomaterials for SCI regeneration. BDNF; Brain derived neurotrophic factor, BMS; Basso mouse scale, HyA; Hyaluronic Acid, NT3; Neurotrophin 3.

Natural Biomaterials and LV Therapy for SCI				
Biomaterial	Therapeutic Strategy	Injury Model	Results	Ref
Agarose	Agarose channel scaffold with LV-NT3 injected rostral to implantation site.	Implanted immediately after C4–5 2 mm long section removal in rat.	LV-NT3 + scaffolds had a significantly higher number of axons exiting the scaffold -vs- scaffold alone and injected LV-NT3 transduced autologous stromal cells. Leptomeningeal fibroblastic scar found at both ends of scaffold and impeded re-penetration of axons into white and grey matter	[75]
HyA	HyA-PEG microspheres mixed with PLL and LV-BDNF or LV-NT3 before injection.	Injected immediately after spinal cord T8-T10 clip compression injury in mice.	LV-BDNF scaffolds had significantly more axons within scaffold -vs- LV-NT3 but not compared to control no LV scaffolds 8 weeks post injury. LV-BDNF scaffolds had significantly more myelinated fibres within scaffold -vs- LV-NT3 and control scaffolds 8 weeks post injury. LV-BDNF scaffolds showed trend increase in BMS hind limb function throughout (no score increase in other treatment groups).	[72]

**Table 7:** Studies combining LVs with Synthetic biomaterials for SCI regeneration. BBS; Berg Balance Scale, MBP; myelin basic protein, NF200; Neuron filament protein 200, PEG. Polyethylene (glycol), PF-127; Pluronic F-127, F4/80+ macrophages; pro-regenerative macrophage, IL-10; interleukin-10, PDGF; Platelet derived growth factor, PLG; poly(lactide-co-glycolide), NT3; Neurotrophin 3, Shh; Sonic hedgehog protein.

Synthetic Biomaterials and LV Therapy for SCI				
Biomaterial	Therapeutic Strategy	Injury Model	Results	Ref
PF-127	LV-Lingo1-shRNA mixed with liquid PF127. LV-Lingo-shRNA bolus treatment (with 25% more LV than scaffold +LV).	Implanted immediately after T10 2 mm transection injury in rats.	LV-Lingo1-shRNA + P127-significantly better neuron count, TUNEL doublecortin, NF200, Map-2 and synapsin staining, and BBS scoring -vs- LV-Lingo1 bolus and scaffold alone.	[42]
PEG	LV-Shh loaded PEG sponges pipetted into macropores.	Intrathecal delivery above T9-10 2.25 mm hemi-section immediately or 4 weeks after injury in mice.	Transgene expression persisted up to 8 weeks post-delivery. LV-Shh sponges delivered both chronically and acutely had increased Olig2+ and MBP/NF200 co-staining along with decreased GFAP+ staining in comparison to control sponges at 8 weeks post implant.	[17]
	PEG hydrogel tubes with LV-IL10 injected directly into tubes before implantation.	Mouse C5 1.15-mm lateral hemisection.	LV-IL-10 scaffolds showed trend increase in % M2 macrophages -vs- control scaffolds. No difference in myelinated fibres or neuronal ingrowth in LV-IL10 loaded and control.	[65]
PLG	Multi-channel PLG bridge with LV-NT3 or LV-BDNF pipetted into channels.	Implanted immediately after T9-10 4 mm hemi-section in rat.	LV-NT3 and LV-BDNF scaffolds had ~2-fold significant increase in no. of axons at rostral end of scaffold at 4 weeks -vs- control scaffold. LV-NT3 and LV-BDNF scaffolds had a significant increase in myelinated axons -vs- control scaffold. No significant change in macrophage infiltration between all groups. No evidence of fibroblastic scar formation. CSPG staining peaked at 1–2 week post implant and then declined.	[18,78]
	Multi-channel PLG bridge with heparin coating loaded with LV-Shh.	Implanted immediately after T9-10 2.25 mm hemi-section in rats.	Surface modification did not affect extent of axon growth into bridge, or myelination. LV-Shh scaffolds had significantly higher MBP+ cells in bridge -vs- control scaffold 8 weeks post injury.	[46]
	Multi-channel PLG bridge loaded with LV-Noggin and/or LV-PDGF.	Implanted immediately after C5 1.5 mm hemi-section in rats.	No significant difference in number of axons or myelination between all groups LV-PDGF + LV-Noggin Scaffolds showed a significant increase in BMS open field scores from 4 weeks onward and in myelination 8 weeks post implant -vs- control scaffold.	[77]
	Multi-channel PLG bridge loaded with LV-IL10, LV-NT3 or LVs with a polycistronic mRNA encoding both IL-10 and NT-3 (LV-IL10-NT3).	Implanted immediately after T9-10 2.5 mm hemisection in rats.	LV-IL10, LV-NT3 and LV-IL10-NT3 scaffolds had significantly better motor scores on the ladder beam -vs- control scaffold. LV-IL10-NT3 had significantly better motor scores -vs- all other groups on week 12. LV-IL10 scaffolds had the highest significant F4/80+ staining -vs- all groups. LV-IL10 scaffolds- significantly higher no. myelinated fibres -vs- control and LV-IL10-NT3 scaffolds. LV-IL10-NT3 scaffolds had significantly higher F4/80+ staining and myelinated fibres -vs- control and LV-NT3 groups. LV-IL10-NT3 showed a significant increase in axon density -vs- all other groups. LV-IL10 and LV-IL10-NT3 showed a significant decrease in cold sensitivity -vs- all other groups	[76]

## Data Availability

No new data were created or analyzed in this study. Data sharing is not applicable to this article.
